# Supplementary material for: Effect of Egg Washing and Hen Age on Cuticle Quality and Bacterial Adherence in Table Eggs
Source: Microorganisms. 2024 Oct 8;12(10):2027. doi: 10.3390/microorganisms12102027 (PMC11509795; doi:10.3390/microorganisms12102027)
Supplement: Supplementary file 1 [file microorganisms-12-02027-s001.zip › microorganisms-3225752-supplementary.pdf]

Supplemental Information

```
Red=1;
Green=2;

imageExtension = ".czi";
myImagePath = getDirectory("Input directory");
imageList = getFileList(myImagePath);


for (i=0; i<imageList.length; i++) {
   if (endsWith(imageList[i], imageExtension)) {
     fileInfo = myImagePath + imageList[i];
     print("Opening \"" + myImagePath + imageList[i] + "\"");
     run("Bio-Formats Importer", "open=[" + fileInfo + "] color_mode=Colorized view=Hyperstack stack_order=XYCZT");
         run("Z Project...", "projection=[Sum Slices]");
{
     Stack.setActiveChannels(Red);
         run("Measure");
     print("Done Red");

}
{
     Stack.setChannel(Green);
         run("Measure");
     print("Done Green");

}
close();
   }
}
print("Finished");
```

Supplemental Figure S1. An in-house script containing image acquisition code for processing of background fluorescence to estimate cuticle.

```
Red=1;
Green=2;

imageExtension = ".czi";
myImagePath = getDirectory("Input directory");
imageList = getFileList(myImagePath);


for (i=0; i<imageList.length; i++) {
   if (endsWith(imageList[i], imageExtension)) {
     fileInfo = myImagePath + imageList[i];
     print("Opening \"" + myImagePath + imageList[i] + "\"");
     run("Bio-Formats Importer", "open=[" + fileInfo + "] color_mode=Default rois_import=[ROI manager] split_channels view=Hyperstack stack_order=XYCZT");
{
     Stack.setChannel(Green);
     run("Z Project...", "projection=[Sum Slices]");
         run("Subtract Background...", "rolling=5");
     setAutoThreshold("Yen dark");
     setOption("BlackBackground", true);
     run("Create Selection");
     run("Measure");


}
{
        id = getImageID();
    title = getTitle();
    dotIndex = indexOf(title, ".");
    basename = substring(title, 0, dotIndex);
    path = myImagePath;
    selectImage(id);
    tileTitle = basename + "mask";
    run("Duplicate...", "title=" + tileTitle);
        selectWindow(title);
        run("Create Mask");
    saveAs("tiff",path+tileTitle);
    close();
}
 while (nImages>0) {
          selectImage(nImages);
          close();
      }
   }
}
print("Finished");
```

Supplemental Figure S2. An in-house script containing image acquisition code for processing of bacterial fluorescence to estimate bacterial adherence.
